# Biofilm production by *Haemophilus influenzae* and *Streptococcus pneumoniae* isolated from the nasopharynx of children with acute otitis media

**DOI:** 10.1186/s12879-018-3657-9

**Published:** 2019-01-11

**Authors:** Quentin Vermee, Robert Cohen, Constantin Hays, Emmanuelle Varon, Stephane Bonacorsi, Stephane Bechet, Franck Thollot, François Corrard, Claire Poyart, Corinne Levy, Josette Raymond

**Affiliations:** 1 Bactériologie, Hôpital Cochin, Université Paris Descartes, 27 rue du Faubourg Saint Jacques, 75679 Paris Cedex 14, France; 2 Association Clinique et Thérapeutique Infantile du Val de Marne (ACTIV), 94 Saint-Maur des Fossés, France; 3 IMRB- GRC GEMINI, Clinical Research Center (CRC), Centre Hospitalier Intercommunal de Créteil, Université Paris Est, Créteil, France; 4 Service de Néonatologie, Centre Hospitalier Intercommunal de Créteil, Créteil, France; 5 Bactériologie, Hôpital Georges Pompidou, Université Paris Descartes, Paris, France; 6 Bactériologie, Hôpital Robert Debré, Université Diderot, Paris, France; 7 Essey les Nancy, AFPA, Paris, France

**Keywords:** *Haemophilus influenzae*, *Streptococcus pneumoniae*, Biofilm, AOM, Naspharynx

## Abstract

**Background:**

Biofilm production by *Haemophilus influenzae* and *Streptococcus pneumoniae* has been implicated in the pathogenesis of otitis media, mainly in chronic and recurrent cases. We studied the *“*in vitro*”* biofilm production by these 2 species isolated alone or together from the nasopharynx of children with acute otitis media.

**Methods:**

The studied strains were from 3 pneumococcal conjugate vaccine (PCV) periods: pre-PCV7, post-PCV7/pre-PCV13 and post-PCV13. A modified microtiter plate assay with crystal violet stain was used to study the biofilm production of 182 *H. influenzae* and 191 *S. pneumonia*e strains.

**Results:**

Overall, 117/181 (64.6%) *H. influenzae* and 128/191 (66.8%) *S. pneumoniae* strains produced biofilm. The proportion of biofilm-producing *H. influenzae* strains was greater with than without the isolation of *S. pneumoniae* in the same sample (75.5% vs 52.3%, *p* = 0.001). Conversely, the proportion of biofilm-producing *S. pneumoniae* strains was not affected by the presence or not of *H. influenzae* (66.3% vs 67.4%). *S. pneumoniae* serotypes 6B, 15B/C, 19A, 35F and 35B were the better biofilm producers (80%). Serotypes 11A, 14, 15A, 19F and 19A were more associated with *H. influenzae* biofilm-producing strains. Overall, 89/94 (94.6%) of cases with combined isolation showed biofilm production by *S. pneumoniae* or *H. influenzae*.

**Conclusion:**

This study emphasizes the high proportion of biofilm production by *H. influenzae* and *S. pneumoniae* strains isolated from the nasopharynx of children with acute otitis media, which reinforces the results of studies suggesting the importance of biofilm in the pathogenesis of acute otitis media.

**Electronic supplementary material:**

The online version of this article (10.1186/s12879-018-3657-9) contains supplementary material, which is available to authorized users.

## Background

Acute otitis media (AOM) is the leading cause of bacterial infections in childhood, about 700 million cases each year, and the leading cause of antibiotic prescription [1]. More than 80% of children have at least one episode of AOM before age 4 years and 40% will have 6 or more recurrences by age 7 years [2, 3]. Otitis media is a multifactorial disease, from uncomplicated AOM to more complex recurrent and chronic cases. Most cases of AOM resolve spontaneously, but complications that can occur include suppurative ones and long-term effects such as hearing loss.

The major pathogens involved in AOM, also called “otopathogens”, are *Streptococcus pneumoniae* and non-typable *Haemophilus influenzae* (NT-*Hi*) (Casey et al., 2004, Block et al., 2004) as well as *Moraxella catarrhalis* [4, 5]. NT-*Hi* is frequently associated with AOM treatment failure, recurrence and otitis media with effusion [6, 7].

The role of biofilm production has been suggested in several types of AOM. A biofilm is dynamic multimicrobial community adhering to a surface and enclosed in a matrix rich in exopolysaccharides, proteins, and nucleic acids. Bacteria living inside this structure are protected against external aggression such as the host immune system and antibiotic treatment [8]. In vivo biofilm production may involve multiple bacteria species, and several studies involving dual-species biofilm experiments have suggested complex inter-species interactions [9–11]. Some studies suggest that the persistence of bacterial species and notably NT-*Hi* in a biofilm-structured community plays a role in the pathogenesis of chronic, recurrent or non-responsive otitis media. Indeed, because of inefficient clearance of bacteria from the middle ear, the biofilm acts as a pathogen reservoir [12–16]. In situ hybridization or immunohistology techniques are probably the best methods to detect biofilm in these cases.

In the opposite spectrum of OM diseases, oto-pathogens are present in nasopharyngeal (NP) flora in biofilm, and some authors suggest that AOM episodes occur when bacteria escape from the biofilm surface to the surrounding space, in the region of adenoids, then Eustachian tube dysfunction promotes the penetration of the strains in the middle ear [17]. In these cases, hybridization and/or immunohistology techniques cannot be used, and other methods, animal models or in vitro methods, are needed to assess the role of biofilm. The capacity for NT*-Hi* and *S. pneumoniae* to produce biofilm can be demonstrated in vitro or in vivo. In vivo studies require a Chinchilla model of otitis media or direct detection by confocal laser scanning microscopy [18–22]. In vitro studies, such as microtiter plates or continuous-culture once-through flow cell, were described in several studies [14, 23–25]. Methods based on PCR techniques have been described, but they cannot discriminate between planktonic and biofilm-growing bacteria [26]. In our previous study, by using a modification of the microtiter plate assay with crystal violet (CV) stain, we found that 49% of *H. influenzae* strains isolated from children’s nasopharynx produced biofilm [27].

Because of the pain caused by tympanocentesis, most clinical-practice guidelines for AOM generally do not recommend taking bacteriological samples of middle ear fluid except for treatment-failure and recalcitrant cases [28]. Kaur et al. used multilocus sequence typing to compare strains isolated from NP and middle-ear fluid samples in 34 children during an AOM episode and found the same sequence type of NT-*Hi* in 31/34 children (84%), which highlighted the similarity of strains isolated from both sites [28]. NP colonization with potential middle-ear pathogens is considered the initial event leading to AOM in humans, frequently preceded by or associated with viral infection [29, 30]. Furthermore, some studies suggested that biofilm production ability differs by pneumococcal serotype [24, 25, 31, 32].

Here, we evaluated biofilm production by NT*-Hi* and *S. pneumoniae* strains isolated alone or together in the nasopharynx of the same patient and analyze biofilm production according to serotypes.

## Patients and methods

### Patients

NT-*Hi* and *S. pneumoniae* strains were isolated from NP samples from children with a diagnosis of AOM according to the Paradise criteria i.e. fever with or without otalgia and associated with bulging tympanic membrane and inflammation signs [33]. Because tympanocentesis is not a recommended first-line method for diagnosis of AOM, we used NP samples from patients with AOM and stringent criteria for the diagnosis of otitis. Bacterial strains were isolated from children included in the following 2 studies that used the same inclusion criteria.

Before PCV7 implementation, we performed 4 randomized trials of antibiotic treatment for AOM. These studies used standardized protocols: the same inclusion criteria for AOM, same age of enrolled children and bacteriological NP samples at enrolment to assess the NP oto-pathogen carriage [34]. These studies included 1807 children. After PCV implementation (PCV7 then PCV13), we investigated otopathogen carriage in 4405 children with AOM from 2001 to 2014 [35]. In both studies, the following data were collected: sex, age, history of AOM, antibiotic treatment during the previous 3 months, daycare attendance modality, vaccination status, and clinical symptoms (temperature > 38.5 °C, otalgia and/or conjunctivitis). Finally, 1779 children came from unpublished studies with the same design.

#### Establishment of patient groups

To study biofilm production, we randomly selected, for each PCV period, 182 NT-*Hi* strains (64 in pre-PCV7, 56 in post-PCV7/pre-PCV13, 61 in post-PCV13; 1 strain not evaluated) and 191 *S. pneumoniae* strains (64 in pre-PCV7, 64 in post-PCV7/pre-PCV13, 63 in post-PCV13) isolated from 277 of the 9798 children included in the 2 studies. For each PCV period, we defined 4 groups: NT-*Hi* strains isolated alone (*n* = 86; group 1) or with *S. pneumoniae* (*n* = 96; group 2), and *S. pneumoniae* strains isolated alone (*n* = 95; group 3) or with NT*-Hi* (n = 96; group 4). Each group included 24 to 32 strains from the 3 periods. In the groups 1 and 2, we looked for the production of biofilm by *H. influenzae* and in the groups 2 and 4, we looked for the production of biofilm by *S. pneumoniae.*

#### Ethical consent

The protocols were approved by the Saint Germain en Laye Hospital Ethics Committee. Written informed consent was obtained from parents or legal representatives of children.

### Methods

Samples were taken as previously described [34, 35] stored at ambient temperature and arrived within 48 h at the Centre National de Référence des Pneumocoques (Hôpital Européen Georges Pompidou, Paris, France) and the Robert Debré Hospital Bacteriological Laboratory (Paris, France).

Culture was performed according to the same method as previously [34, 35]. Identification was confirmed by matrix-assisted laser desorption ionization-time of flight mass spectrometry (MALDI-TOF MS, Bruker Daltonics, Germany) for *H. influenzae* and MALDI-TOF MS and optochin susceptibility for *S. pneumoniae*.

*S. pneumoniae* serotyping was performed at the French National Reference Center for *S. pneumoniae* (CNRP, Hôpital Européen Georges Pompidou, Paris, France) [34, 35].

Antibiotic susceptibility of *S. pneumoniae* and *H. influenzae* was determined according to t those proposed by the CASFM/EUCAST (www.sfm-microbiologie.org/UserFiles/files/casfm/CASFM2016_V1_0_FEVRIER.pdf).

#### Microtiter biofilm formation assay

Biofilm production was evaluated by a modification of the microtiter plate assay with crystal violet (CV) stain as described for NT-*Hi* [27]. This assay was based on the ability of bacteria to adhere to solid polystyrene surfaces by using biofilm. The biofilm production assay was adapted for *S. pneumoniae*. Briefly, *S. pneumoniae* isolates were sub-cultured on 5% blood agar (Biomérieux, La Balme Les Grottes, France) and incubated at 37 °C for 24 h under 5% CO_2_. Then, *S. pneumoniae* isolates were grown for 8 h at 35 °C with 5% CO_2_ in BHI (Becton Dickinson, Le Pont-De-Claix, France). Thereafter, the protocol was identical to that for NT-*Hi*. For each strain, the assay was performed in triplicate and repeat in 2 independent experiments.

A *Staphylococcus aureus* strain known as a strong biofilm producer was used as a positive control in each experiment and a *Streptococcus agalactiae* strain known as a poor biofilm producer was used as a negative control. These strains were kindly provided by the Streptococci national reference center (Pr C. Poyart, Cochin Hospital, Paris, France). For each assay, the BHI was tested alone to calculate the biofilm formation index (BFI).

#### Determination of cutoff values

The biofilm formation index (BFI) was determined by applying 3 different formulas. The first was derived from Kadurugamuwa et al. [36]. The authors calculated the BFI at optical density 595 nm as BFI = AB – CW, where AB represents stained wells containing attached bacteria and CW, stained control wells containing bacteria-free medium only, here supplemented with BHI. The second method, described by Soto et al. [37], used the same index with a different formula: BFI = AB/CW. The third method was according to Niu et al. (2004): BFI = (AB – CW)/G, where G is bacterial planktonic growth control [38]. The assays were performed in triplicate. Finally, the results for each method were studied by terciles to classify the biofilm production semi-quantitatively into 3 categories: strong production (S), moderate production (M) and absence of production (negative; N) according to the cutoff values proposed by Naves et al. [39]. However, unlike Naves et al., we grouped the strains with moderate or low biofilm production [39].

Naves et al. showed that biofilm production is strongly altered by culture conditions, environmental factors and methodology. For these reasons and to avoid these pitfalls, we combined the 3 different BFI calculation methods. This method of calculation was validated to study the biofilm production by *H. influenza*e and published in 2014 [27].

#### Statistical analysis

Double data entry involved use of the software 4D v12.5. Statistical analysis involved use of Stata SE 13.1 (Stata Corp., College Station, TX, USA). Pearson chi-square test or Fisher exact test was used to compare groups (two-tailed tests). Potential risk factors were identified by univariate analysis (*p* < 0.25) and introduced in multivariate logistic regression models. Only significant risk factors (*p* < 0.05) remained in the final model. For statistical analysis, we merged strong and moderate biofilm-producing bacteria to compare 2 groups: “moderate or strong biofilm-producer” versus “low biofilm-producer” for both NT*-Hi* and *S. pneumoniae*. *P* < 0.05 was considered statistically significant.

## Results

### Methods for calculating the biofilm production

The cut-offs used to determine the intensity of biofilm production in isolates of *S. pneumoniae* and *H. influenzae* were as followed where AB represented the stained wells containing attached bacteria, CW the stained control wells containing bacteria-free medium only and G the bacterial growth control i) AB – CW: strong production > 0.30, moderate production 0.10–0.30, negative < 0.10 ii) AB / CW: strong production > 6, moderate production 2–6, negative < 2 iii)[(AB – CW) / G]: strong production > 1.10, moderate production 0.35–1.10, negative < 0.35.

Strains were classified according to agreement of at least 2 of the 3 methods used to calculate biofilm production by *S. pneumoniae* (Additional file 1: Supplementary Data S1) and *H. influenzae* (Additional file 2: Supplementary Data S2). Among 182 NT*-Hi* strains, 62 (34%) were classified as strong biofilm producers, 55 (30.2%) moderate producers and 64 (35.1%) non-producers. One strain could not be evaluated. Overall, 117/181 (64.6%) NT-*Hi* strains produced biofilm (Table 1). By using the same classification, among the 191 *S. pneumoniae* strains, 63 (33%) were classified as strong biofilm producers, 64 (33.5%) moderate producers and 63 (33%) non-producers. Overall, 128/191 (66.8%) *S. pneumoniae* strains produced biofilm (Table 1).

**Table 1:** Biofilm production by *H. influenza*e and *S. pneumoniae* isolated alone or together by pneumococcal conjugate vaccine (PCV) period

Biofilm production	Pre-PCV7	Post-PCV7/Pre-PCV13	Post-PCV13	Total
*H. influenzae*				
Isolated alone	19/32 (59.4%)	15/24 (62.5%) ^a^	11/30 (36.7%) ^a^	45/86 (52.3%) ^c^
Isolated with *S. pneumoniae*	24/32 (75%)	28/32 (87.5%) ^b^	20/32 (63.3%) ^b^	72/96 (75.5%) ^c^
Total	43/64 (67.2%)	43/56 (76.8%)	31/61 (50.8%)	117/181 (64.6%)
*S. pneumoniae*				
Isolated alone	21/32 (65.6%)	22/32 (68.8%)	21/31 (67.7%)	64/95 (67.4%)
Isolated with *H. influenzae*	23/32 (71.9%)	21/32 (64.5%)	20/32 (62.5%)	64/96 (66.3%)
Total	44/64 (68.8%)	43/64 (66.7%)	41/63 (65.1%)	128/191 (66.8%)

^a^ *H. influenzae* isolated alone between the post-PCV7/prePCV13 and post-PCV13 periods *p* = 0.05

^b^ *H. influenzae* associated with *S. pneumoniae* between the post-PCV7/pre-PCV13 and post-PCV13 periods *p* = 0.02

^c^ Total of *H. influenzae* alone or associated with S*. pneumoniae* independent of period *p* = 0.001

### Evolution of the proportion of biofilm-producing strains

Between the pre-PCV7 and post-PCV7/pre-PCV13 periods, the proportion of NT-*Hi* biofilm-producing strains was stable (67.2 and 76.8%, respectively, p = ns), whether isolated alone or with *S. pneumoniae* strains (Table 2). However, the proportion of NT-*Hi* biofilm-producing strains was lower in the post-PCV13 than post-PCV7/pre-PCV13 period for strains isolated alone (62.5 and 36.7%, respectively, *p* = 0.05) or with *S. pneumoniae* strains (87.5 and 63.3%, respectively, *p* = 0.02). Overall, the proportion of NT*-Hi* biofilm-producing strains was greater when isolated with *S. pneumoniae* (75.5% vs 52.3%, *p* = 0.001) (Table 2).

**Table 2:** Serotypes of *S. pneumoniae* isolates producing biofilm, isolated alone or with *H. influenzae*

		*S. pneumoniae*						*H. influenzae*	Biofilm production by either *S. pneumoniae* or *H. influenzae* isolated together
	Biofilm production by *S. pneumoniae* Total	Biofilm production by *S. pneumoniae* isolated alone	Biofilm production by *S. pneumoniae* isolated with *H. influenzae*					Biofilm production by *H. influenzae* isolated with *S. pneumoniae*	
			*S. pneumoniae* Total	*S. pneumoniae* Biofilm+		*S. pneumoniae* Biofilm -			
Serotype (*n*)	*n* (%)	*n* (%)	Biofilm + *n* (%)	Hi Biofilm +	Hi Biofilm -	Hi Biofilm +	Hi Biofilm -	*n* (%)	*n* (%)
PCV7									
6B (11)	10 (91)	7/8 (88)	3/3 (100)	1	2			1/3(33)	3/3 (100)
9 V (2)	2 (100)	1/1 (100)	1/1 (100)	1				1/1 (100)	1/1 (100)
14 (10)	6(60)	1/2 (50)	5/8 (63)	5		3		8/8 (100)	8/8 (100)
18C (1)	1 (100)	0	1/1 (100)	1				1/1 (100)	1/1 (100)
19F (17)	7 (41)	2/6 (33)	5/11 (45)	4	1	4	2	8/11 (73)	9/11 (82)
23F (13)	5 (38)	3 /7(43)	2/5 (40)	1	1	2	1	3/5 (60)	4/5 (80)
Total	31/54 (57.4)	14/24 (58.3)	17/29 (58.6)	13	4	9	3	22/29 (75.9)	26/29 (89.9)
PCV13 additional serotypes									
1 (1)	0	0	0/1			1		1/1 (100)	1/1(100)
3 (1)	0	0/1	0/0					0	0
5 (1)	1 (100)	0	1/1 (100)		1			0/1	1/1 (100)
6A (6)	3 (50)	2/5 (40)	1/1 (100)	1				1/1(100)	1/1 (100)
7F (2)	2 (100)	1/1 (100)	1/1 (100)	1				1/1(100)	1/1 (100)
19A (17)	16 (94)	8/8 (100)	8/9 (89)	6	2	1		7/9 (78)	9/9 (100)
Total	22/28 (78.5)	11/15 (73.3)	11/13(84)	8	3	2		10/13 (77)	13/13 (100)
Other serotypes									
15B/C (15)	14 (93)	8/8 (100)	6/7 (86)	4	2		1	4/7 (57)	6/7 (86)
15A (12)	6 (50)	3/5 (60)	3/7 (43)	3		4		7/7 (100)	7/7 (100)
11A (9)	6 (67)	3/3 (100)	3/6 (50)	3		2	1	5/6 (83)	5/6 (83)
23B (9)	3 (33)	0/4	3/5 (60)	3		2		5/5 (100)	5/5 (100)
23A (12)	8 (67)	6/8 (75)	2/4 (50)	1	1	2		2/3 (67)	4/4 (100)
10A (6)	3 (50)	0/2	3/4 (75)	1	2	1		2/3 (67)	4/4 (100)
21 (5)	2 (40)	1/2 (50)	1/3 (33)		1	2		2/3(67)	3/3 (100)
35F (4)	4 (100)	1/1 (100)	3/3 (100)	2	1			2/3 (67)	3/3 (100)
35B (6)	5 (83)	3/4 (75)	2/2 (100)		2			0/2	2/2 (100)
6C (4)	2 (50)	1/2 (50)	1/2 (50)	1		1		2/2 (100)	2/2 (100)
17F (4)	4 (100)	3/3 (100)	1/1 (100)	1				1/1(100)	1/1 (100)
22F(2), 29(2)	2 (50)	0/2	2/2 (100)	1				2/2 (100)	2/2(100)
24F (2)	1 (50)	1/1 (100)	0/1				1	0/1	0
15F (1)	0 (0)	0/1 (0)	0/1			1		1/1 (100)	1/1 (100)
16(1)	1 (100)	0	1/1 (100)	1				1/1 (100)	1/1 (100)
38 (1)	1 (100)	0	1/1 (100)		1			0/1	1/ 1(100)
8(2), 12F (1), 25A (1) 31 (1), 33F (1)	5 (83.3)	5/6 (83.3)	0/0					0	0
Total	67/101 (69.3)	43/62 (69.3)	36/57 (63.1)	24	11	17	4	36/48 (75)	46/48 (94.6)
*NT* (7)	7 (100)	3/3(100)	4/4 (100)	3	1			3/4 (75)	4/4 (100)
Total	127/190 (66.8%)	64/95 (67.4%)	64/97 (66%)	46/65 (71%)	18/65 (27.6%)	26/32 (81.2%)	6/32 (18.7%)	71/94 (75.5%)	**89/94 (94.6%)**

PCV7: Serotypes included in the PCV7 vaccin

PCV13 additionnal serotypes: Additionnal serotypes added in the PCV13 vaccin

NT: non typable serotypes

Conversely, the proportion of *S. pneumoniae* biofilm-producing strains did not change over the study periods, whether isolated alone (65.6 and 67.7% in pre-PCV7 and post-PCV13 periods) or with NT*-Hi* strains (71.9 and 62.5% in pre-PCV7 and post-PCV13 periods). Overall, the proportion of *S. pneumoniae* biofilm-producing strains did not differ whether isolated alone or with NT-*Hi* strains [67.4% vs. 66.3%, *p* = 0.92] (Table 2).

### Characteristics of S. pneumoniae biofilm-producing strains

We found no differences in biofilm-producing ability by pneumococcal serotype (Table 2). The proportion of *S. pneumoniae* serotypes included in PCV7 (54/191) that produced biofilm was similar to that for other serotypes: 57.4% (31/54) versus 70.6% (96/137), *p* = 0.09 (data not shown). Moreover, the proportion did not differ between the 6 additional *S. pneumoniae* serotypes included in PCV13 and other serotypes [78.5% (22/28) vs 64.4% (105/163), *p* = 0.14)]. Strains of serotypes 6B, 15B/C, 19A, 35F, and 35B produced biofilm in more than 80% of the cases. In contrast, strains of serotypes 23B, 23F,19F were the lowest producers (40% of strains) (Table 2).

The *S. pneumoniae* serotypes frequently isolated with NT*-Hi* strains were 11A, 14, 15A, 15B/C, 19A, 19F, 23F and 23B, representing 60.4% (58/96) of the combination. *H. influenzae* biofilm-producing strains were isolated more often with serotypes 11A, 14, 15A, 19F and 19A. This association did not affect biofilm production by *S. pneumoniae*.

Susceptibility or resistance to penicillin did not differ with and without biofilm production: 64.2% (61/96) versus 69.5% (66/95), *p* = 0.44.

Overall, 75.5% (72/96) of NT*-Hi* strains produced biofilm when isolated with *S. pneumoniae* strains. Serotypes of *S. pneumoniae* were not associated with biofilm production by NT*-Hi* strains.

When isolated from the same nasopharynx, *S. pneumoniae* or *H. influenzae* or both produced biofilm in 94.6% (89/94) of cases (Table 3).

**Table 3:** Demographic characteristics and clinical signs of children according to the biofilm production

Characteristics and clinical signs	Biofilm production by *H. influenzae*	p*	Biofilm production by *S. pneumoniae*	p*
Day-care center	47/72 (65.3)	0.66	42/63 (66.7)	0.99
Recurrent acute otitis media	22/33 (66.7)	0.77	19/29 (65.5)	0.87
Conjunctivitis	59/97 (60.8)	0.27	42/66 (63.6)	0.52
Otalgia	96/145 (66.2)	0.26	105/156 (67.3)	0.68
Temperature > 38.5 °C	42/56 (75.0)	**0.047**	45/71 (63.4)	0.46
Fever + otalgia	50/67 (74.6)	**0.025**	58/86 (67.4)	0.87
Antibiotics 3 months before enrollment	53/87 (60.9)	0.34	55/82 (67.1)	0.95

*p comparing biofilm strain producers and non-producers

### Relation between clinical signs and biofilm production

Table 3 presents the demographic characteristics and clinical signs by biofilm production. Biofilm was produced significantly more often by NT-*Hi* strains isolated from children with than without fever (temperature > 38.5 °C) or fever + otalgia [75% (42/56) vs 59.7% (74/124), *p* = 0.047 and 74.6% (50/67) vs 56.5% (65/112), *p* = 0.025]. These relations were not observed for *S. pneumoniae* (*p* = 0.46 and *p* = 0.87, respectively).

### Antimicrobial susceptibility and serotyping of the strains

The proportion of resistant strains significantly decreased from the pre-PCV7 to post-PCV13 periods for both NT*-Hi* and *S. pneumoniae*. Among NT*-Hi* strains, the proportion of β-lactamase–producing strains decreased from 51.5% (33/64) in the pre-PCV7 period to 17.8% (10/56) and 17.7% (11/62) in the post-PCV7/pre-PCV13 and post-PCV13 periods, respectively (*p* < 0.001). As well, the proportion of *S. pneumoniae* strains with decreased susceptibility to penicillin decreased from 65.7% in the pre-PCV7 period to 45.4% (29/64) and 39.7% (25/63) in the post-PCV7/pre-PCV13 and post-PCV13 periods (*p* = 0.003). Among *S. pneumoniae* strains, the proportion of those with erythromycin susceptibility increased from 46.8% (30/64) to 81% (51/63) between the post-PCV7/pre-PCV13 and post-PCV13 periods, respectively (p < 0.001).

After PCV implementation, the distribution of the S*. pneumoniae* serotypes changed according to the study period (Additional file 3: Supplementary Data S3). In the pre-PCV7 period, the serotypes of 47/64 *S. pneumoniae* strains were included in PCV7, 10/64 involved the 6 additional serotypes included in PCV13, and 3 were serotype 15B/C. In the post-PCV7/pre-PCV13 period, the serotypes of 6/64 *S. pneumoniae* strains were included in PCV7, 19/64 involved the 6 additional serotypes included in PCV13, and 38/62 were of various serotypes. In the post-PCV13 period, only 1/63 serotypes was included in PCV13.

## Discussion

To our knowledge, it is the first study exploring in a large cohort the production of biofilms of *H. influenzae* and *S. pneumoniae* isolated alone or together from NP flora of children with AOM. Previously, the role of biofilm production in AOM was mainly explored for *S. pneumoniae* [25, 31, 40] or *H. influenzae* [22, 27, 41] or both in Chinchilla models [42].

We used here three methods of calculation because there is no reference method for biofilm production. We found high biofilm production for both *S. pneumoniae* and *H. influenzae* (66.8 and 64.4%, respectively) with no variation in proportion for *S. pneumoniae* over PCV implementation periods. Of note, the proportion of NT-*Hi* biofilm-producing strains was greater when *H. influenzae* strains were isolated with *S. pneumoniae*, which agreed with the results of Hong et al. [10].

When *S. pneumoniae* and *H. influenzae* were isolated together, 93.7% of cases showed biofilm production by *S. pneumoniae* and/or *H. influenzae*. Residence of a bacterium within a biofilm allows for global changes in gene and protein expression profiles, which has many effects on cell physiology, promoting adhesion and cohesion properties of biofilm cells, thereby increasing its persistence [43]. Recent studies demonstrated that *H. influenzae* and *S. pneumoniae* modulate the expression of each other’s virulence genes, which results in persistent biofilm, mainly by upregulati type IV pilus structural protein (*pilA*) by *H. influenzae*, thereby playing an important role in adhesion and biofilm stability [9, 12]. Hong et al. reported significantly downregulated expression of pneumococcal genes regulating autolysis and fratricide, *lytA* and *cbpD*, on co-culture with NT*-Hi*, which suggests that pneumococcal survival and biofilm production can be enhanced in the presence of NT-*Hi* [10] .

We found that S*. pneumoniae* serotypes 6B, 15B/C, 19A, 35F and 35B were the best biofilm producers. Previously, serotypes 14, 6B, 15B/C, and 11A were found efficient in producing biofilm [25, 40], whereas Domenech et al., reported serotypes 35B and 11A, 19A as efficient [24]. Therefore, these results support the validity of our method because the 5 serotypes we found as the best biofilm producers were those previously described. Analysis of a large database of AOM serotypes revealed that serotype 19A has the highest disease potential for AOM [44]. The high production of biofilm may be one explanation for this phenomenon.

Our study has several limitations. The first is the lack of direct evaluation of biofilm in biological samples in middle ear fluid (MEF) with alternative methods such as confocal microscopy after live/dead staining [45, 46]. However, we assumed that this method could not be used for a large population with AOM. Second, one can argue that the studied strains were isolated from the nasopharynx and not from MEF. We postulate, as do other authors, that the reservoir of bacterial species implicated in AOM is the nasopharynx, and their carriage precedes AOM [29, 30]. Previously we demonstrated no significant difference in biofilm production between NT-*Hi* strains from MEF and NP samples [27]. In a previous prospective study, Bingen et al., using pulsed-field gel electrophoresis in conjunctivitis-otitis syndrome*,* revealed identical NT*-Hi* strains isolated from MEF and conjunctivitis tissue [47] . Van Dongen et al., in a systematic review found NT-*Hi* strains isolated from both MEF and NP samples in 80% of cases [48]. More recently, Van Hoecke et al., investigating the presence of otopathogenic bacteria in middle ear effusion and adenoids of children with chronic otitis, found *NT-Hi* and *S. pneumoniae*, isolated from both locations, genetically identical in 13/14 cases [22]. The third limitation is that we could have used knock-out mutants as a control. However, many genes appear to be involved, although the role of each appears to vary when biofilm is produced in batch or continuous culture. Proteomic studies have revealed an increase in number of proteins synthesized de novo and differences in protein production patterns during *S. pneumoniae* biofilm development [23]. In these conditions, in vivo studies are difficult. Another limitation is that these isolates came only from children with AOM and not from healthy children or children with chronic AOM. However, these results support the hypothesis that multispecies biofilm is the basis for the chronicity of otitis media as previously suggested [13]. Finally, even if a larger number of strains would have allowed us to have more statistical power, our sample allowed us to detect some statistical differences.

The last limitation of our study is that biofilm production was studied in monoculture because of the different culture requirements of the 2 strains. One reason was the difficulty in co-cultivating the 2 bacteria because of the rapid lysis of *S. pneumoniae* in a liquid medium. The incubation time required for the experiment was not the same for both bacteria.

The role of biofilm in otitis media is not fully understood, and we lack a universally accepted or feasible method to study biofilm formation in vitro and in vivo in humans. We are aware that we are simply bringing some modest information to a complex puzzle. However, if our study has several limitations, it has also several strengths as follows. 1) To our knowledge, it is the first study exploring in a large cohort the production of biofilm of *H. influenzae* and *S. pneumoniae* isolated from NP flora of children with AOM. 2) We were able to demonstrate H. *influenzae* and/or *S. pneumoniae* biofilm production in all the clinical situations and more particularly by NT-*Hi* strains isolated from children with fever or fever associated with otalgia; this seems an important point reinforcing biofilm production as a ubiquitous phenomenon in carriage. 3) More than 60% of *S. pneumoniae* and *H. influenzae* strains produced biofilm, and this proportion increased significantly for *H. influenzae* when the 2 bacterial species were isolated in the same sample. Furthermore, biofilm production globally did not differ by period or vaccine and non-vaccine type. 4) Our results agree with those of previous studies regarding the ability of some *S. pneumoniae* serotypes to produce biofilm.

## Conclusion

This study highlighted the biofilm production among the 2 main oto-pathogens strains (93.7% of cases showed biofilm production by *S. pneumoniae* or *H. influenzae*). The proportion of NT*-Hi* biofilm-producing strains was greater when isolated with *S. pneumoniae.* Furthermore, some *S. pneumoniae* serotypes produced more biofilm than did other.

## Additional files

Additional file 1: Supplementary Data S1: Different cut-offs used to determine the biofilm production by *S. pneumoniae:* the different cut-offs used were as followed where AB represented the stained wells containing attached bacteria, CW the stained control wells containing bacteria-free medium only and G the bacterial growth control i) AB – CW: strong production > 0.30, moderate production 0.10–0.30, negative < 0.10 ii) AB / CW: strong production > 6, moderate production 2–6, negative < 2 iii)[(AB – CW) / G]: strong production > 1.10, moderate production 0.35–1.10, negative < 0.35. Strains were classified according to agreement of at least 2 of the 3 methods used to calculate biofilm production. (XLS 195 kb)

Additional file 2: Supplementary Data S2: Different cut-offs used to determine the biofilm production by *H. influenzae:* the different cut-offs used were as followed where AB represented the stained wells containing attached bacteria, CW the stained control wells containing bacteria-free medium only and G the bacterial growth control i) AB – CW: strong production > 0.30, moderate production 0.10–0.30, negative < 0.10 ii) AB / CW: strong production > 6, moderate production 2–6, negative < 2 iii)[(AB – CW) / G]: strong production > 1.10, moderate production 0.35–1.10, negative < 0.35. Strains were classified according to agreement of at least 2 of the 3 methods used to calculate biofilm production. (XLSX 76 kb)

Additional file 3: Supplementary Data S3: Distribution of S*. pneumoniae* serotypes by study period (pre-PCV7, post-PCV7/pre-PCV13 and post-PCV13). (DOCX 15 kb)

## Abbreviations

AOM: Acute otitis media
BFI: Biofilm formation index
BHI: Brain heart infusion
CASFM/EUCAST: Comité de l’Antibiogramme de la Société Française de Microbiologie/European Committee on Antimicrobial Susceptibility Testing
CNRP: Centre National de Référence des Pneumocoques
CV: Crystal violet
MALDI-TOF MS: Matrix-assisted laser desorption ionization-time of flight mass spectrometry
MEF: Middle ear fluid
NP: Nasopharyngeal
NT-*Hi*: *Haemophilus influenzae*
PCR: Polymerase chain reaction
PCV: Pneumococcal conjugate Vaccine
