# Supplementary material for: Biofilm production by Haemophilus influenzae and Streptococcus pneumoniae isolated from the nasopharynx of children with acute otitis media
Source: BMC Infect Dis. 2019 Jan 11;19:44. doi: 10.1186/s12879-018-3657-9 (PMC6329076; doi:10.1186/s12879-018-3657-9)
Supplement: Supplementary file 3 — Supplementary Data S3: Distribution of S. pneumoniae serotypes by study period (pre-PCV7, post-PCV7/pre-PCV13 and post-PCV13). (DOCX 15 kb) [file 12879_2018_3657_MOESM3_ESM.docx]

**Supplementary data S1**: Distribution of S*. pneumoniae* serotypes by study period (pre-PCV7, post-PCV7/pre-PCV13 and post-PCV13).

|  | Pre-PCV7 Period | Post-PCV7/ Pre-PCV13 Period | Post-PCV13 Period |
| --- | --- | --- | --- |
| **PCV7 serotypes (n)** |  |  |  |
| 14 | **10** | - | - |
| 18C | **1** | - | - |
| 19F | **10** | 6 | 1 |
| 23F | **13** | - | - |
| 6B | **11** | - | - |
| 9V | **2** | - | - |
| **Additional PCV13 serotypes** | |  |  |
| 1 | 1 | - | - |
| 19A | **6** | **12** | - |
| 3 | 1 | - | - |
| 5 | 1 | - | - |
| 6A | 1 | 5 | - |
| 7F | - | 2 | - |
| **Other serotypes** |  |  |  |
| 6C | - | 4 | - |
| 8 | - | 1 | 1 |
| 10A | - | 1 | **5** |
| 11A | - | 2 | **7** |
| 12F | - | - | 1 |
| 15A | - | 6 | **6** |
| 15B/C | 3 | 3 | **9** |
| 15F |  | 1 | - |
| 16 | - | - | 1 |
| 17F | - | 4 | - |
| 21 | - | - | 5 |
| 22F | - | 1 | 1 |
| 23A | - | 4 | **8** |
| 23B | - | 4 | **5** |
| 24F | - | 1 | 1 |
| 25A | - | 1 | - |
| 29 | - | - | 2 |
| 31 | - | 1 | - |
| 33F | - | - | 1 |
| 35B | - | 1 | **5** |
| 35F | - | 1 | 3 |
| 38 | - | - | 1 |
| Non-typable | 4 | 3 | - |
